# From Clinical Trials to the Front Line: Vinflunine for Treatment of Urothelial Cell Carcinoma at the National Cancer Institute of Naples

**DOI:** 10.3389/fphar.2016.00110

**Published:** 2016-05-03

**Authors:** Gaetano Facchini, Chiara Della Pepa, Carla Cavaliere, Sabrina C. Cecere, Marilena Di Napoli, Carmine D'Aniello, Anna Crispo, Gelsomina Iovane, Piera Maiolino, Teresa Tramontano, Raffaele Piscitelli, Salvatore Pisconti, Maurizio Montella, Massimiliano Berretta, Domenico Sorrentino, Sisto Perdonà, Sandro Pignata

**Affiliations:** ^1^Division of MedicalOncology, Department of Uro-Gynaecological Oncology, Istituto Nazionale Tumori Fondazione G. PascaleNaples, Italy; ^2^Department of Onco-Ematology Medical Oncology, S.G. Moscati Hospital of TarantoTaranto, Italy; ^3^Unit of Epidemiology, Struttura Complessa di Statistica Medica, Biometria e Bioinformatica, Fondazione Istituto Nazionale TumoriNaples, Italy; ^4^Pharmacy Unit, Istituto Nazionale Tumori, Istituto Nazionale Tumori-Fondazione G. PascaleNaples, Italy; ^5^Division of Medical Oncology–CROAviano, Italy; ^6^Division of Urology, Department of Uro-Gynaecological Oncology, Istituto Nazionale Tumori Fondazione G. PascaleNaples, Italy

**Keywords:** vinflunine, transitional cell cancer of the urothelial tract, response rate, progression free survival, overall survival

## Abstract

**Background:** The efficacy of Vinflunine, after failure of platinum-based chemotherapy in patients with metastatic or recurrent Transitional Cell Cancer of the Urothelial Tract, TCCU, has been demonstrated in an international, randomized, phase III trial comparing Vinflunine plus Best Supportive Care, BSC, with BSC alone. On the basis of that study vinflunine has been approved by the European Medicine Association, EMA, for treatment of TCCU patients after failure of a platinum treatment. However, since data in clinical trials often differ from routine clinical practice due to unselected population and less strict monitoring, “real life” experiences are very helpful to verify the efficacy of a new therapy.

**Methods:** This was a spontaneous, observational, retrospective study involving 43 patients with metastatic TCCU treated with vinflunine at our cancer center, data about demographics, disease characteristics, and previous treatments were collected and outcome and toxicities of vinflunine were analyzed.

**Results:** 41 of 43 patients were eligible for RR analysis, the Overall RR was 12%, the Disease Control Rate was 29%; when including only patients treated in II line the DCR rose to 33%; the median PFS and the median OS were 2.2 and 6.9 months, respectively.

**Conclusion:** Our findings were consistent with the outcome data emerged in the phase III randomized trial and in the other observational studies conducted all around Europe in the last 2–3 years. This experience supports the use of vinflunine in patients with advanced TTCU as effective and manageable antineoplastic drug.

## Introduction

Transitional cell cancer of the urothelial tract, TCCU, is the sixth most common type of cancer in western countries (Siegel et al., 2013; Franco et al., 2014), in most cases it involves the bladder but may also origin from the ureter or the renal pelvis; the estimated number of deaths from bladder cancer in US in 2015 are 16.000 which means that the need of new therapeutic approaches is extremely urgent (Bladder cancer incidence and mortality National Cancer Institute^1^; Leopardo et al., 2013). Advanced TCCU is considered a relatively chemosensitive tumor due to the high Response Rate, RR, observed in first line with platinum-based regimens, varying from 40 to 70% (Von der Maase et al., 2005; Roberts et al., 2006; Bellmunt et al., 2012; Ferro et al., 2012; Marra et al., 2013; Cavaliere et al., 2014), nevertheless the duration of response is limited and when progression after primary treatment occurs the outcome is generally poor (Iaffaioli et al., 1997; Strocchi et al., 2004). Several regimens have been tested in the recurrent setting, including both single agents (Albers et al., 2002; Vaughn et al., 2002; Franco et al., 2011) and combinations (Bellmunt et al., 2002; Iaffaioli et al., 2006; Lin et al., 2007; Marra et al., 2008) but they showed modest activity often associated with significant toxicity. Interesting results in patients progressed after platinum were reported with the gemcitabine-paclitaxel doublet which demonstrated relevant activity in two phase II trials (Sternberg et al., 2001; Reimann et al., 2006) however, up to very recently, a standard second line treatment did not exist. Vinflunine is an antineoplastic agent belonging to the vinca alkaloids family, such drugs act inducing apoptosis by prevention of microtubule assembly during mitosis (Aparicio et al., 2012). The most important advantage of this novel molecule, when compared with the other agents of the same class, is the higher inhibition of microtubules dynamics thus strongly acting on mitotic spindle rather than on the axonal tubuline, vinflunine exposes patients to a minor risk of neurotoxicity (Kruczynski and Hill, 2001; Braguer et al., 2008).

The efficacy of Vinflunine after failure of platinum-based chemotherapy has been proved in two phase II trials involving a cohort of 51 and 175 patients respectively (Culine et al., 2006; Vaughn et al., 2009). In the work by Culine et al. (2006) the reported Overall Response Rate, ORR, was 18% (95% Confidence Interval, CI: 8.4–30.9%) and the Disease Control Rate (Partial Response, PR, plus Stable Disease, SD), DCR, was 67% (95%CI: 52.1–79.3%); the median duration of response was 9.1 months (95% CI: 4.2–15.0); the median Progression Free Survival, PFS, was 3.0 months (95% CI: 2.4–3.8); the median Overall Survival, OS, was 6.6 months (95% CI: 4.8–7.6; Culine et al., 2006). In the second phase II study, by Vaughn et al, the observed RR with Vinflunine was 15% (95% CI, 9–21%), the median duration of response was 6.0 months; the authors reported a median PFS of 2.8 months and a median OS of 8.2 months (Vaughn et al., 2009). In both the phase II clinical trials the side effects were manageable and myelosuppression was the main issue followed by constipation and fatigue (Culine et al., 2006; Vaughn et al., 2009).

The encouraging results reported in such studies led to an international, randomized, phase III trial comparing Vinflunine plus Best Supportive Care, BSC, with BSC alone (Bellmunt et al., 2009). Bellmunt et al. showed that Vinflunine significantly improved PFS (3 vs. 1.5 months, *P* = 0.0012); ORR (16 vs. 0%, *P* = 0063); and DCR (41.1 vs. 24.8%, *P* = 0.0063). The primary endpoint of the study was a 2 months advantage in the OS, this was achieved (6.9 vs. 4.6) but was not statistically significant (*p* = 0.29) in the Intention To Treat, ITT, population (*n* = 365) while it was confirmed (6.9 vs. 4.3) and reached the statistical significance (*p* = 0.04) in the eligible population (*n* = 357). These data were then confirmed at a long-term follow up at more than 3, 5 years (Bellmunt et al., 2013). Vinflunine was the first drug, in September 2009, receiving the approval from the European Medicine Association, EMA, for treatment of TCCU patients after failure of a platinum-based regimen (European Medicine Agency^2^).

Usually the experience in clinical practice significantly differs from the trials findings mainly because of the minor patients selection and the lack of a so strict patients monitoring, observational studies run in routine clinics may help to provide a more realistic scenario though they have several limits related to the number of subjects, the often retrospective design and the potential statistical bias.

We retrospectively reviewed our experience with Vinflunine in the treatment of advanced TCCU.

## Patients and methods

This was a spontaneous retrospective study, approved by the local ethic committee, looking at patients with metastatic TCCU treated with Vinflunine at the National Cancer Institute, Giovanni Pascale Foundation, in Naples, Italy. Inclusion criteria included: age > 18 years old, histologically-proven diagnosis of TCCU, stage IV disease, measurable lesions at the CT scan, prior or current treatment with Vinflunine (any line), signed informed consent (if patient had not deceased). Retrieving from our archives the data about metastatic TTCU patients, we found 43 patients who have received Vinflunine from February 2012 to March 2015 in either second or subsequent lines.

As baseline characteristics we evaluated demographics and previous treatment data, we also analyzed several risk factors of interest such as ECOG (Eastern Cooperative Oncology Group) Performance Status, PS, renal function, anemia and visceral involvement and their effect on survival. In terms of outcome we described Response Rate, RR (either overall and II vs. subsequent lines), the Progression Free Survival, PFS, and the OS, Overall Survival.

We closed the data collection on 10th March 2015, estimation of likelihood events for PFS and OS were calculated according to the Kaplan-Meier method, statistical differences between curves were calculated using log-rank test. The Cox proportional hazards model was used to test the effect of the considered risk factors on survival in multivariate analyses; Hazard Ratios (HRs) and 95% Confidence Interval (CIs) were estimated, adjusting for variables that were significant at univariate analysis; a *p* < 0.05 was considered significant. Statistical analysis was performed using SPSS (version 21; SPSS, Inc., Chicago, IL).

## Results

### Baseline characteristics

As previously mentioned we enrolled 43 subjects, 40 patients were male (93%), median age was 63, 5 (range 41–76), 34 patients (79%) had bladder urothelial carcinoma while 9 (21%) had other TCCU. As to treatment 25 patients (58%) have had surgery; 11 patients (26%) have received a perioperative treatment which mainly consisted of adjuvant chemotherapy. As to previous regimens, all patients undergone a first line chemotherapy prior to Vinflunine, 56%with carboplatin, 37% with cisplatin, 7% with gemcitabine and paclitaxel. Though a small number of patients, 8 (19%) received Vinflunine as III or IV line the most of our cohort were given Vinflunine as II line; patients experienced relapse/progression less than 6 months after completion of the prior chemotherapy in 74% of cases.

We focused on several prognostic factors to verify their impact on survival: (i) anemia; (ii) poor PS; (iii) visceral involvement, and (iv) compromised renal function were individuated as potentially relevant risk factors. Anemia, defined as hemoglobin < 10 mg/dL, poor PS, and liver metastases were demonstrated to be significant prognostic factors by Sonpavde et al., however, compared to their model, we considered “poor” PS ≥ 2 rather than > 0 and “presence of liver metastases” was replaced by “visceral involvement” (Sonpavde et al., 2013). Though reduced renal function failed to predict survival in TCCU patients in previous papers (Bellmunt et al., 2010; Galsky et al., 2012) we decided to include such parameter, defined as Creatinine Clearance < 40 ml/min in our analysis in order to confirm or deny this lack of association.

We found that, when starting Vinflunine, 10% of our patients had a PS ≥ 2; 40% had a Creatinine Clearance < 60 ml/min (5% < 40 ml/min); 60% had lung or liver metastases; 12% had grade 2 anemia (Hemoglobin, Hb, < 10 mg/dl). Globally at least one risk factor was observed in more than half of the cases while about the 20% presented 2 or more of them.

The baseline characteristics of our cohort are summarized in Tables 1A,B, the former showing demographics and previous treatment data, the latter explaining population features in terms of prognostic risk factors.

**Table 1A:** **Baseline characteristics: demographics and previous treatment**.

	***n =* 43 (%)**
**AGE**	
Median	63,5
Range	41–76
**SEX**	
Male	40 (93)
Female	3 (7)
**ORIGIN**	
Bladder	34 (79)
Other	9 (21)
**SURGERY**	
Not received	18 (42)
Cistectomy	22 (51)
Nephroureterectomy	3 (7)
**PERIOPERATIVE TREATMENT**	
Not received	32 (74)
Neoadjuvant	0 (0)
Adjuvant	11 (26)
CT only	7 (16)
RT only	2 (5)
CT + RT	2 (5)
**FIRST-LINE REGIMEN**	
CBDCA	24 (56)
CDDP	16 (37)
GEM/PTX	3 (7)
**VINFLUNINE LINE**	
II line	35 (81)
III line	6 (14)
IV line	2 (5)
**TIME TO RELAPSE/PROGRESSION AFTER THE PRIOR CHEMOTHERAPY**	
≤ 6 months	32 (74)
>6 months	11 (26)

*CT, Chemotherapy; RT, Radiotherapy; CBDCA, Carboplatin; CDDP, Cisplatin; GEM/PTX, Gemcitabine/Paclitaxel*.

**Table 1B:** **Baseline characteristics: Risk factors**.

	***n =* 43 (%)**
**PS**	
0	3 (7)
1	30 (70)
≥2	10 (23)
**CREATININE CLEARANCE (mL/min)**	
>60	26 (60)
40–60	15 (35)
<40	2 (5)
**VISCERAL INVOLVEMENT**	
No	17 (40)
Yes	26 (60)
**Hgb level mg/dL**	
≥10	38 (88)
<10	5 (12)
**RISK FACTORS STRATIFICATION**	
0	9 (21)
1	25 (58)
≥2	9 (21)

*PS, Performance Status*.

### Outcome

Forty-one of Forty-three patients were eligible for RR analysis (two patients excluded due to unavailability of post-treatment imaging), the Overall RR was 12%, the Disease Control Rate, DCR (PR + SD) was 29%. When including only patients treated in II line the DCR rose to 33%.

On 10th March 2015, 38 of our 43 patients had progressed (median follow up 24 months) hence were evaluable for PFS, the median PFS resulted 2.2 months in the entire cohort and 7.2 months in the 12 patients who have had PR or SD as best response to Vinflunine (DCR group).

Looking at the survival outcome we found a median OS of 6.9 months; when restricting the evaluation to the 35 patients receiving Vinflunine in II line the value reached the median of 11.8 months.

All the outcome findings are shown in Table 2.

**Table 2:** **Outcome**.

**RR (Overall)**	***n* = 41 (%)**
PR	5 (12)
SD	7 (17)
DCR	12 (29)
**RR (II line)**	***n* = 33 (%)**
PR	4 (12)
SD	7 (21)
DCR	11 (33)
**RR (III and IV line)**	***n* = 8 (%)**
PR	1 (13)
SD	0 (0)
DCR	1 (13)
**PFS (months)**	
mPFS	*n* = 38
	2.2
mPFS (DCR pts)	*n* = 12
	7.2
**OS (months)**		
mOS (Overall)	*n* = 43
	6.9
mOS (VIN II line)	*n* = 35
	11.8
mOS (VIN III–IV line)	*n* = 8
	5.6

*RR, Response Rate; PR, Partial Response; SD, Stable Disease; DCR, Disease Control (PR+SD); PFS, Progression Free Survival; mPFS, median PFS; OS, Overall Survival; mOS, median OS; VIN, Vinflunine*.

**Figure 1:**
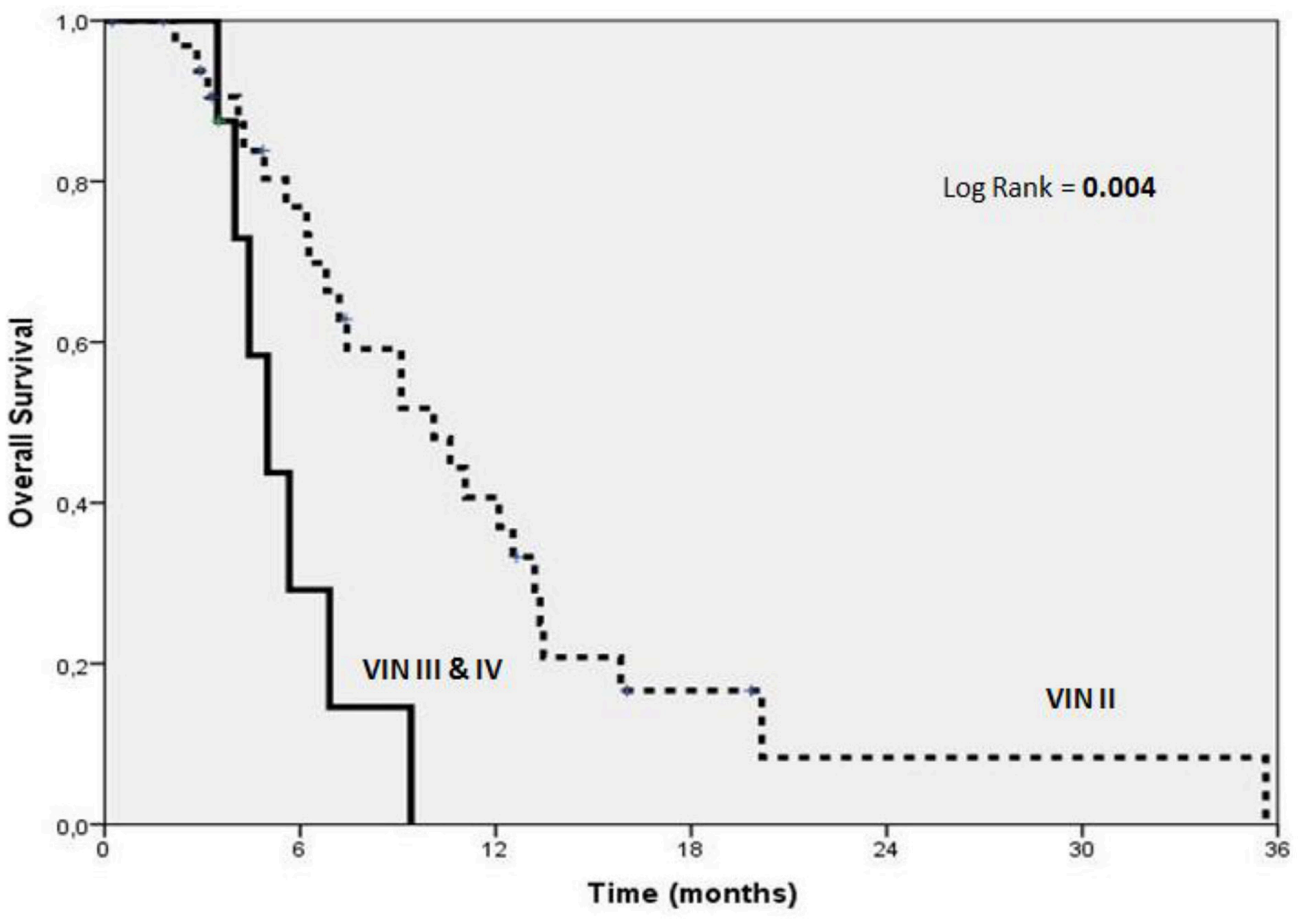
**Kaplan-Meier estimates of OS according to VINFLUNINE lines**.

As documented in previous works (Bellmunt et al., 2010; Sonpavde et al., 2013), we found that anemia had an effect on survival with an HR of 3.4 (*p* = 0.01), on the other side, in contrast with data in literature (Bellmunt et al., 2010; Galsky et al., 2012), we observed that altered renal function had an effect on survival (HR 3, *p* = 0.05) while PS did not (HR 2.6 *p* = n.s.), however, due to the limited patients cohort, these findings need to be carefully interpreted (Table 3); also the treatment with Vinflunine in II rather than in III or IV line showed to have an effect on survival (Figure 1).

**Table 3:** **Risk of death based on risk factors analysis**.

**Risk factor**	**HR**	**95% C.I**.	***p***
Hb < 10 g/dL	3.4	1.26–9.1	0.01
Creatinine Cl < 40 ml/min	3.0	1.0–9.3	0.05
PS ≥ 2	2.6	0.6–12.3	0.2
Visceral involvement	1.9	0.77–4.9	0.1
≥2 risk factors	2.6	1.1–5.9	0.002

*HR, Hazard Ratio; C.I. Confidence Interval; Hb, Haemoglobin; Creatinine Cl, Creatinine Clearance; PS, Performance Status*.

### Safety

In our experience Vinflunine appeared as a very manageable therapy, there were no major safety issues and no treatment discontinuations due to toxicity (Table 4). The most frequent non hematological side effects were fatigue (51%), constipation (26%), and abdominal pain (12%) which were grade 3/4 only in two patients. In regards to mielotoxicity we observed grade 3–4 neutropenia in the 19% of patients and two cases, 5%, of febrile neutropenia (both spontaneously recovered). Infusion/injection site reactions occur in 2 of 43 patients (5%), however roughly 90% of the patients included in this analysis had a venous catheter either PICC-line or PORT-A-CATH.

**Table 4:** **Safety**.

**Adverse event**	**Any grade *n* (%)**	**Grade 1/3 *n* (%)**
**Fatigue/asthenia**	**22 (51)**	1 (2)
Nausea	15 (35)	0 (0)
Vomiting	8 (19)	0 (0)
Stomatitis/mucositis	4 (9)	0 (0)
**Abdominal pain**	**5 (12)**	0 (0)
**Costipation**	**11 (26)**	1 (2)
Myalgia	3 (7)	0 (0)
Neuropathy sensory	3 (7)	0 (0)
Alopecia	5 (12)	0 (0)
Infusion/injection site reaction	**2 (5)**	0 (0)
**Anemia**	**18 (42)**	2 (5)
**Neutropenia**	**31 (72)**	**8 (19)**
Febrile neutropenia	**2 (5)**	**2 (5)**
**Thrombocytopenia**	**11 (26)**	2 (5)

*The bold values highlight the most frequent side effects*.

## Discussion

The introduction of a new treatment into the routine clinical practice usually reveals new aspects in regards to both outcome and safety, with innovative and expensive therapies constantly emerging, the issue of the cost-effectiveness ratio become every day more important and the Healthcare institutions are understandably more and more cautious about budget resources. The observational, non-interventional studies, are crucial to clarify the real impact of a new treatment on the affected population and to provide useful information about cost, side effects, and tolerability in unselected cohorts.

With more than half of our patients presenting with 1 unfavorable risk factor and 20% having 2 or more of them we can say that this was a very “unselected” population however our efficacy findings appear comparable with the outcome observed in the phase III randomized trial (Bellmunt et al., 2009), also the other “real life” experiences (Hegele et al., 2013; Medioni et al., 2013; Castellano et al., 2014; Palacka et al., 2014; Hussain et al., 2015; Moriceau et al., 2015; Retz et al., 2015), which have been conducted all around Europe, in the last 2–3 years, showed equivalent data (see Table 5).

**Table 5:** **Vinflunine for metastatic/recurrent TTCU: the phase III trial compared to real-life**.

**References**	**Country**	***n***	**Vinflunine line**	**RR**	**DCR**	**mPFS**	**mOS**
Medioni et al., ECCO, 2013	France	134	II	22	51	4.2	8.2
Castellano et al., BMC, 2014	Spain	102	II	24.5	65.7	3.9	10
Palacka et al., Klin Onkol, 2014	Slovak Republic	16	II	13.3	–	2.3	5.2
Hegele et al., Urol Int, 2014	Germany	21	II	19.1	47.7	4.4	6.2
Hussain et al., ASCO GU, 2015	UK	37	II	32	52.6	–	9.5
Moriceau et al., Clin Genit, 2015	France	19	II (47%) III or more (53%)	32	53	2.9	5.6
Retz et al., BMC, 2015	Germany	77	II (66%) I (12%) III or more (22%)	23.4	53.2	–	7.7
Bellmunt et al., JCO, 2009	Europe	253	II	8.6	41	3	6.9
Facchini et al.	Italy	43	II (81%) III or more (19%)	12	29	2.2	6.9

This study confirmed that Vinflunine is a very manageable antineoplastic drug, effective in a setting in which there are very few therapeutic options and patients clinical conditions are often deteriorated due to bulky abdominal disease and poor renal function.

The available retrospective analyses together with our work demonstrate that clinicians can feel confident in administering Vinflunine to unselected patients; fatigue, constipation, and abdominal pain are the most frequent non-hematologic side effects. As to mielotoxicity grade 3–4 neutropenia was reported in 50% of patients in the phase III trial and only in 19% of our cohort, however, due to the retrospective nature of this analysis adverse events may have been somehow underestimated.

The strengths of this study include the number of patients, which is more than acceptable considering that this was a single center experience and Vinflunine became available in Italy only very recently, and the data completeness which consented to make a reliable efficacy statistical analysis. On the other hand our feeling is that the hematology and biochemistry side effects may have been underestimated due to the retrospective nature and that including patients treated in III or subsequent line may have altered the cohort homogeneity.

## Conclusion

To the best of our knowledge this is the first work focused on Italian patients treated with Vinflunine, all the European literature support the use of this drug after failure of a previous platinum treatment in patients with advanced TTCU and our study reported outcome data consistent with the findings of the other “real life” trials and not very distant from the randomized phase III trial which included a much more selected population.

## Author contributions

GF, CP, CC, SC, MD, CD, SPi design the study and wrote the manuscript; AC, MM performed the statistical analysis; GI, LP, MB, DS, SPe, SPig performed the data collection; PM, TT, and RP performed the pharmacoeconomical analysis.

### Conflict of interest statement

The authors declare that the research was conducted in the absence of any commercial or financial relationships that could be construed as a potential conflict of interest.

## Footnotes

^1^http://www.cancer.gov/cancertopics/pdq/treatment/bladder/HealthProfessional/page1.

^2^http://www.ema.europa.eu/ema/index.jsp?curl=pages/medicines/human/medicines/000983/human_med_000866.jsp&mid=WC0b01ac058001d124.
